# Factor Structure of the Japanese Version of the Edinburgh Postnatal Depression Scale in the Postpartum Period

**DOI:** 10.1371/journal.pone.0103941

**Published:** 2014-08-04

**Authors:** Chika Kubota, Takashi Okada, Branko Aleksic, Yukako Nakamura, Shohko Kunimoto, Mako Morikawa, Tomoko Shiino, Ai Tamaji, Harue Ohoka, Naomi Banno, Tokiko Morita, Satomi Murase, Setsuko Goto, Atsuko Kanai, Tomoko Masuda, Masahiko Ando, Norio Ozaki

**Affiliations:** 1 Department of Psychiatry, Nagoya University Graduate School of Medicine, Nagoya, Aichi, Japan; 2 Nihon Fukushi University Chuo College of Social Services, Nagoya, Aichi, Japan; 3 Meijo University Graduate School of Pharmaceutical Sciences, Nagoya, Aichi, Japan; 4 Liaison Medical Marunouchi, Nagoya, Aichi, Japan; 5 Nagoya University Graduate School of Medicine, Nagoya, Aichi, Japan; 6 Graduate School of Education and Human Development, Nagoya University, Nagoya, Aichi, Japan; 7 Graduate School of Law, Nagoya University, Nagoya, Aichi, Japan; 8 Center for Advanced Medicine and Clinical Research, Nagoya University Graduate School of Medicine, Nagoya, Aichi, Japan; University of Pennsylvania, United States of America

## Abstract

**Background:**

The Edinburgh Postnatal Depression Scale (EPDS) is a widely used screening tool for postpartum depression (PPD). Although the reliability and validity of EPDS in Japanese has been confirmed and the prevalence of PPD is found to be about the same as Western countries, the factor structure of the Japanese version of EPDS has not been elucidated yet.

**Methods:**

690 Japanese mothers completed all items of the EPDS at 1 month postpartum. We divided them randomly into two sample sets. The first sample set (n = 345) was used for exploratory factor analysis, and the second sample set was used (n = 345) for confirmatory factor analysis.

**Results:**

The result of exploratory factor analysis indicated a three-factor model consisting of anxiety, depression and anhedonia. The results of confirmatory factor analysis suggested that the anxiety and anhedonia factors existed for EPDS in a sample of Japanese women at 1 month postpartum. The depression factor varies by the models of acceptable fit.

**Conclusions:**

We examined EPDS scores. As a result, “anxiety” and “anhedonia” exist for EPDS among postpartum women in Japan as already reported in Western countries. Cross-cultural research is needed for future research.

## Introduction

Postpartum depression (PPD) is a type of major depressive disorder after childbirth and is distinguished from maternity blues in terms of onset, severity and duration of symptoms. The prevalence of PPD is estimated at approximately 13% from meta-analysis [1], [2]. Our study shows 10.4% of women in Japan experienced depressive symptomatology assessed by the Edinburgh Postnatal Depression Scale (EPDS) [3]. PPD is a major mental health problem in women with children [4]. First, PPD reduces maternal mental health and quality of life. 5–14% of perinatal and postnatal women have thoughts of self-harm, and suicides account for up to 20% of postpartum deaths [5]. Second, PPD has a negative influence on child health and development [6], because it interferes with the mother's ability to care for a baby and handle other daily tasks. Third, the mother-child relationship often worsens because of PPD [8]. Severe depression is also reported to be associated with child abuse [9].

Early detection and intervention are essential for maternal and child health. EPDS, a 10-item self-administered questionnaire for early detection of PPD [10], has been the most widely used screening tool for PPD across countries and cultures. In recent studies, the factor structure of the original English version of EPDS has been reported as shown in Table 1 [11]–[18]. These results suggest that anxiety symptoms account for a significant part of PPD symptoms, unlike typical major depressive disorders. There are only a few studies about the factor structure of EPDS outside Western countries, but these studies show similar results: that EPDS was found to contain at least two factors, a depressive factor and an anxiety factor in Brazil [19], China [19], and the Netherlands [19].

**Table 1:** Factor structure of the English version of the EPDS.

First author, Publishied year	Period	Country	N	Method	Rotation	Factor structure		
						Factor 1	Factor 2	Factor 3
Tuohy & McVey,2008	Postpartum 6.47 months	U.K.	440	EEA	Oblimin	Non-specific depressive symptoms:7, 8, 9, 10	Anhedonia:1, 2	Anxiety symptoms:3, 4, 5
King, 2012	Postpartum 1 week-12 months	U.S.A.	169	CFA	None	Non-specific depressive symptoms:7, 8, 9, 10	Anhedonia:1, 2	Anxiety symptoms:3, 4, 5
Astbury, 1994	Postpartum 8–9 months	Australia	790	PCA	Oblimin	Depression:1, 2, 6, 7, 8, 9, 10	Anxiety:3, 4, 5	-
Matthey, 2008	Postpartum 6 weeks	Australia	238	PCA	Varimax	Depression:1, 2, 6, 7, 8, 9, 10	Anxiety:3, 4, 5	-
Phillips, 2009	Postpartum 0–12 months	Australia	309	EEA/CFA	Oblimin	Depression:1, 2, 6, 7, 8, 9, 10	Anxiety:3, 4, 5	-
Ross, 2003	Pregnant 36 weeks/Postpartum 6 and 16 weeks	Canada	150	PCA	Varimax	Depression:1, 2, 8, 9	Anxiety:3, 4, 5	Suicide 10
Jomeen, 2005	Postpartum 13.6 weeks	U.K.	101	EEA/CFA	Oblimin	Depression:1, 2, 6, 7, 8, 9	Anxiety:3, 4, 5	Suicide 10
Swalm, 2010	Postpartum 26 weeks	Australia	4706	PCA	Varimax	Anhedonia:1, 2	Anxiety:3, 4, 5	-

(EEA: exploratory factor analysis, CFA: confirmatory factor analysis, PCA: principal component analysis).

The pathology of PPD has been thought to be caused by biological and psychosocial changes with pregnancy and childbirth. There is no direct evidence that PPD has a common pathology across different populations, ethnicities and cultures; however, the commonality of the prevalence of PPD [20] supports this idea. If a common pathophysiology can be proven and this hypothesis supported, it will become a significant step towards understanding the common pathology of PPD. Because the cross-cultural consistency of the factor structure of EPDS, however, has yet not been examined, particularly outside Western countries, more research is needed to answer the question.

In Japan, the reliability and validity of EPDS in Japanese has been confirmed and the prevalence of PPD is found to be comparable to the Western countries, but the factor structure of the Japanese version of EPDS has not been elucidated. Therefore, we examined the symptomatological structure of PPD measured with the Japanese version of EPDS to compare with the structure of the original English version of EPDS already reported in Western countries.

## Methods

### Participants

Participants were recruited between August 2004 and October 2012. Every participant was an outpatient in a maternity ward at one of three obstetrics and gynecology hospitals in Nagoya, Japan. The three obstetrics hospitals were a general hospital (Nagoya Teishin Hospital), an obstetrics and gynecology hospital (Kaseki Hospital), and a university hospital (Nagoya University Hospital).

The eligibility criteria were as follows:

1. attending at one of the three hospitals consecutively
2. 20 years of age or older
3. ability to understand the questionnaire written in Japanese.

### Procedure

We explained our research design and methods to pregnant women at maternity programs or outpatient care. In these three hospitals, every outpatient equally receives an orientation for birth hospitalization during the second trimester at outpatient care or maternity program. We matched the timing of the invitation with the timing of the orientation during the second trimester which every patient participates. At the same time, participants received a set of agreement documents and questionnaires. Each woman was asked to participate in the study voluntarily and to answer all of the questions according to the predefined schedule. If she agreed to participate in the study, she was requested to return the two sealed envelopes that contained the anonymous questionnaire and the signed agreement separately. This was to guarantee privacy. We considered a voluntarily returned envelope consent to participate in this research.

### Measurements

We assessed depressive symptoms in participants as well as their social background (i.e. years of schooling, demographic parameters). Depressive symptoms were evaluated by EPDS at about 1 month after childbirth.

EPDS is a 10-item self-report screening tool for postnatal depression. Each item is scored on a 4-point scale ranging from 0 to 3. Total scores can range from 0 to 30. The English version of EPDS has good internal consistency (Cronbach's alpha = 0.87) and reliability (split half reliability = 0.88) [10].

EPDS was translated into Japanese by Okano et al. in 1996 and confirmed that the retranslated English version was the equivalent to the original English version [21]. The validity and reliability of this Japanese version of EPDS were also examined against 115 non-pregnant women and 47 women at 1 month postpartum by Okano et al [21]. It had good internal consistency (Cronbach's alpha  = 0.78) and test–retest reliability (Spearman correlation  = 0.92) [21]. The validity was examined against a diagnosis of major depressive disorder from the semi-structured interview-based Research Diagnostic Criteria (RDC) [22] as external criteria. The total score of the women who have postpartum depression (N = 4) was higher than that of the non-depressive postpartum women (N = 43) [21] and the cut-off point of ≧9 showed good sensitivity (75%) and specificity (93%) [21].

This is the standardized Japanese version and no other Japanese version of EPDS is used in Japan. In this study, we used this Japanese version of EPDS and the cut-off point of ≧9 in accordance with the previous study [21].

### Data analysis

We randomly divided all participants who completed all items of EPDS into two sample sets. The first sample set was used for exploratory factor analysis, and the second sample set for confirmatory factor analysis.

### Exploratory Factor Analysis (EFA)

The number of factors was determined by scree plot. An EFA with maximum-likelihood extraction was undertaken on the full 10-item EPDS. Oblique rotation using the promax rotation was performed due to an expectation that factors would be correlated.

### Confirmatory Factor Analysis (CFA)

We chose the model identified in EFA and the models reported in the original English version of EPDS as follows:

1. Tuohy & McVey/King; three-factor [11], [15]
2. Astbury et al. /Matthey/Phillips et al.; two-factor [13], [16], [17]
3. Ross et al.; three-factor [14]
4. Jomeen et al.; three-factor [12]
5. Swalm et al.; two-factor [18]
6. Model identified in the EFA; three-factor

As recommended for structural equation modeling applications [23], [24], we used the goodness-of-fit index (GFI) [25], adjusted goodness-of-fit index (AGFI) [23], comparative fit index (CFI) [26], and root mean square error of approximation (RMSEA) [27]. A good fit is defined as a GFI greater than 0.95, an AGFI greater than 0.90, a CFI greater than 0.97 and an RMSEA less than 0.05. An acceptable fit is defined as a GFI greater than 0.90, an AGFI greater than 0.85, a CFI greater than 0.95 and an RMSEA less than 0.08 [25] [23] [26] [27]. Data were analyzed using SPSS version 20.0 and Amos 19.0 (IBM Japan, Tokyo, Japan).

## Results

### Characteristics of participants

812 participants agreed to participate in this study. The mean age of the participants was 32.1 years (range: 20 to 45 years, Standard Deviation (S.D.) 4.5 years, interquartile range (IQR) 29–35). Average years of schooling were 14.4 years (range: 9 to 18 years, S.D. = 1.6 years, IQR 14–16). In terms of parity, 67.0% of participants were nulliparous, 24.4% of participants were primiparous, 7.9% of participants had given birth two times, and 0.6% of participants had given birth three times (range: 0 to 3 children, S.D. = 0.7, IQR 0–1). 690 out of the 812 women completed all items of EPDS. The non-response rate is 75 of 812 and the non-valid response rate is 51 of 812.

### EPDS scores

The median postpartum EPDS score was 3 (range: 0–22, S.D. = 4.53, IQR 1–7). Approximately 18.4% of women scored 9–22 and were considered at high risk of postpartum depression. The median infant age was 31.7 days (range: 16–64, S.D. = 6.9 days, IQR 30–34).

### Factor analysis

690 participants who completed all items of EPDS were divided randomly into two groups. The first sample set of 345 participants was used for EFA, and the second sample set of 345 participants was used for CFA.

### EFA

The dataset was found suitable for factor analysis (the Kaiser-Meyer-Olkin index = 0.886). The Cronbach's alpha for the 10-item EPDS was 0.856, indicating the test has good instrument internal reliability. The scree test indicated a three-factor solution which accounted for 64.4% of the variance. The anhedonia, anxiety and depression factors appeared consistent with factors identified in our studies. A coefficient level of 0.45 or above was chosen to indicate significant item factor loading.

The first factor, which explained 43.4% of the total variance, included EPDS items 3, 4, 5 with factor loadings >0.45 (listed in Table 2). Items 3, 4 and 5 were found to have the highest factor loadings (all>0.65), consistent with previous findings that identified this factor as an “anxiety” subscale within EPDS. The second factor explained 12.1% of the total variance, and included items 1 and 2 with factor loadings>0.45. Items 1 and 2 had the highest factor loadings (>0.55), consistent with previous findings that identified this factor as an “anhedonia” subscale within EPDS. The third factor explained 8.8% of the total variance, and included items 7, 8 and 9 with factor loadings >0.45. Items 7 and 9 had the highest factor loadings (>0.7), consistent with previous findings that identified this factor as a “depression” subscale within EPDS.

**Table 2:** Factor analysis of the Japanese version Edinburgh Postnatal Depression Scale.

Items of the EPDS	Factor 1	Factor 2	Factor 3
1. I have been able to laugh and see the funny side of things.	−.034	1.055	−.075
2. I have looked forward with enjoyment to things.	.026	.599	.135
3. I have blamed myself unnecessarily when things went wrong.	.684	.067	.026
4. I have been anxious or worried for no good reason.	.755	.023	−.072
5. I have felt scared or panicky for not very good reason.	.803	−.078	−.044
6. Things have been getting on top of me.	.238	.140	.141
7. I have been so unhappy that I have had difficulty sleeping.	.011	.028	.741
8. I have felt sad or miserable.	.352	.065	.497
9. I have been so unhappy that I have been crying.	−.112	.000	.824
10. The thought of harming myself has occurred to me.	.255	−.086	.267

(N = 690, maximum-likelihood estimation, promax rotation).

### CFA

The goodness-of-fit for the data represented by GFI, AGFI, CFI and RMSEA are shown in Table 3. In the models of Tuohy & McVey and King, GFI and AGFI showed good fit while CFI and RMSEA showed acceptable fit. In the models of Astbury et al. and Matthey and Phillips et al., GFI, AGFI, CFI and RMSEA showed unsatisfactory fit. In the model of Ross et al., GFI, AGFI, CFI and RMSEA showed unsatisfactory fit. In the model of Jomeen et al., GFI, AGFI, CFI and RMSEA showed unsatisfactory fit. In the model of Swalm et al., GFI, AGFI, CFI and RMSEA showed good fit. In the model identified in EFA, GFI and AGFI showed good fit. CFI and RMSEA showed acceptable fit. We therefore concluded that there were four acceptable models: those of Tuohy & McVey, King, Swalm et al., and as identified in EFA. We also concluded that the models of Astbury et al., Matthey and Phillips et al., Ross et al. and Jomeen et al. were unsatisfactory.

**Table 3:** Goodness-of-fit of the models.

First author, Publishied year	Factor structure			Goodness-of-fit of the models			
	Factor 1	Factor 2	Factor 3	GFI	AGFI	CFI	RMSEA
1. Tuohy & McVey, 2008/King, 2012	Non-specific depressive symptoms:7, 8, 9, 10	Anhedonia:1, 2	Anxiety symptoms:3, 4, 5	0.965	0.934	0.958	0.065
2. Astbury, 1994/Matthey, 2008/Phillips, 2009	Depression:1, 2, 6, 7, 8, 9, 10	Anxiety:3, 4, 5	-	0.870	0.796	0.852	0.136
3. Ross, 2003	Depression:1, 2, 8, 9	Anxiety:3, 4, 5	Suicide:10	0.896	0.790	0.881	0.152
4. Jomeen, 2005	Depression:1, 2, 6, 7, 8, 9	Anxiety:3, 4, 5	Suicide:10	0.883	0.810	0.868	0.132
5. Swalm, 2010	Anhedonia:1, 2	Anxiety:3, 4, 5	-	0.992	0.970	0.995	0.05
6. Model identified in the EFA in this study	Anxiety:3, 4, 5	Anhedonia:1, 2	Depression:7, 8, 9	0.954	0.902	0.962	0.092

Correlations between factors in the models of an acceptable fit or a good fit were as follows. In the models of Tuohy & McVey and King, correlation between “anxiety” and “depression” was 0.84, correlation between “depression” and “anhedonia” was 0.64, and correlation between “anhedonia” and “anxiety” was 0.60. In the model of Swalm et al., correlation between “anhedonia” and “anxiety” was 0.60. In the model identified in EFA, correlation between “anxiety” and “depression” was 0.85, correlation between “depression” and “anhedonia” was 0.66, and correlation between “anhedonia” and “anxiety” was 0.60.

## Discussion

This is the first study demonstrating the factor structure of the Japanese version of EPDS using a large sample of postpartum women. The model of EFA reported by Tuohy & McVey, King and Swalm et al., was consistent with our model in the present study of the Japanese version of EPDS. The model consists of common factors, an anxiety factor (items 3, 4 and 5) and an anhedonia factor (items 1 and 2). Thus, our findings suggest that factor structure of EPDS in Japan is basically the same as already reported in Western countries, although there was variance between studies on some items of EPDS.

No previous papers have reported the factor structure of the Japanese version of EPDS, however there are some studies about the symptoms of PPD in Japan. Tamaki et al. showed that women with PPD have strong anxiety symptoms by the State-Trait Anxiety Inventory Trait test [28]. Sato Y et al. revealed that the prevalence of anxiety symptoms was higher than that of depressive symptoms after childbirth [29]. These results also suggest that anxiety symptoms are important to understand the symptomatic character of PPD in Japan.

In the other Asian countries, there are a few studies of the factor structure of EPDS. Small et al. analyzed the factor structure of EPDS in Vietnam, Turkey and Philippines [30].Small et al. pointed out that Item 6 loaded less consistently in the different countries, however they also suggest that EPDS have two or three factors which consists of anxiety and depression. Lau Y et al. showed that EPDS in China consists of the three factors as depression factor(items 6, 7, 8. 9 and 10), anxiety factor(items 3,4 and 5) and anhedonia factor(items 1 and 2) [31]. These results are very similar to the factor structure of EPDS in Japan by our study.

### The depression factor

The depression factor varies across studies. Tuohy & McVey and King suggested items 7, 8, 9, and 10, Swalm suggested no depression factor, and the EFA in our study showed items 7, 8, and 9. Though the best fit was the model of Swalm et al., we proposed the EFA model because the model of Swalm excluded half of all EPDS items. Cross-cultural studies are needed to examine whether a common depression factor exists or not.

### The anhedonia factor

All of the acceptable models show the anhedonia factor (items 1 and 2), which is reverse scoring. As reverse scoring items tend to be in the same cluster [32], we must take into account that reverse scoring items has been found to affect factor analysis.

### The anxiety factor

The anxiety factor (items 3, 4 and 5) was shown in many countries, such as Brazil [33], [34], China [31], and the Netherlands [19]. Considering the existence of a common anxiety factor across different countries and cultures, the importance of anxiety symptoms for PPD has been revealed. In fact, it is reported that about 10% of the women experiencing postpartum depression have anxiety symptoms [35].

The utility of the anxiety factor (items 3, 4 and 5) is discussed in some studies as follows. Some studies have suggested that items 3, 4 and 5 can measure anxiety disorder [18], [36], and other studies suggested that items 3, 4 and 5 are enough for PPD screening [14] [37]. Although the utility of the anxiety factor (item 3, 4 and 5) varies by study, as mentioned before, there is some possibility of common utility of the anxiety factor around the world.

### Correlation between factors

The correlations between “anxiety” and “depression” were found to be high in the models of acceptable fit to the data. These results suggested that there was a very close relationship between depression and anxiety, as previously reported [38], [39], and showed that it was important to focus on anxiety symptoms in PPD screening and care.

### Limitations

There are some types of study bias in this study. First, there is a self-selection bias. They participated in this study voluntarily. This also means that they pay more attention to their mental health. Second, there are losses to follow up. Women with depression are hard to reply the questionnaire. Third, there is a membership bias. We cannot affirm that a standard population in Japan is shown in these participants from three characteristic hospitals, a general hospital, an obstetrical and gynecological hospital, and a university hospital. The patients at the university hospital tended to have pregnancy complications, but these participants accounted for a small percentage of all participants (N = 42, 5.2%). Fourth, there is a non-response bias, however the non-response rate is 75 of 812 and the non-valid response rate is 51 of 812. We consider that these rates are not so high and the result is not affected.

Furthermore, there is a problem with this study design. We did not ask participants for their nationality, citizenship or ethnicity. However, Japan is considered to be highly homogenous in terms of population, therefore we consider this problem does not affect the result of the study.

## Conclusions

We examined factor structure of the Japanese version of EPDS in a large sample size of postpartum women in Japan. As a result, “anxiety”, “depression” and “anhedonia” factors exist for EPDS, as already reported in Western countries. Our findings suggest that the factor structure of EPDS is mostly common across countries and cultures.

### Ethics Statement

This study protocol has been approved by the Ethics Committee of the Nagoya University Graduate School of Medicine.
